# Comparison of Inspecting Non-Ferromagnetic and Ferromagnetic Metals Using Velocity Induced Eddy Current Probe

**DOI:** 10.3390/s18103199

**Published:** 2018-09-21

**Authors:** Bo Feng, Artur L. Ribeiro, Tiago J. Rocha, Helena G. Ramos

**Affiliations:** Instituto de Telecomunicações, Instituto Superior Técnico, Universidade de Lisboa, 1049-001 Lisbon, Portugal; bofeng@tecnico.ulisboa.pt (B.F.); arturlr@ist.utl.pt (A.L.R.); tiago.rocha@tecnico.ulisboa.pt (T.J.R.)

**Keywords:** velocity induced eddy current, non-destructive testing, velocity effect, eddy current testing

## Abstract

A velocity induced eddy current probe has been used to detect cracks in both non-ferromagnetic and ferromagnetic metals. The simulation and experimental results show that this probe can successfully detect cracks in both cases, but further investigation shows that the underlying principles for inspecting non-ferromagnetic and ferromagnetic metals are actually different. For an aluminum plate, the induced eddy current density and the signal amplitude both increase with probe speed, which means the signal is caused by velocity induced eddy currents. For a steel plate, probe speed changes the baselines of the testing signals; however, it has little influence on signal amplitudes. Simulation results show that the signal for cracks in a steel plate is mainly caused by direct magnetic field perturbation rather than velocity induced eddy currents.

## 1. Introduction

Eddy current testing is a technique that has already been successfully used to detect cracks in conductive materials [1,2]. The conventional eddy current testing technique uses a coil driven by alternating current to generate eddy currents inside a specimen. A magnetic field sensor, such as a giant magnetoresistance (GMR) sensor [3], Hall sensor [4,5], or sensing coil [6], is used to measure the magnetic field, which is the vector sum of the magnetic field produced by the current that runs in the excitation coil and the field produced by eddy currents in the specimen. A defect will change the eddy currents’ path and will perturb the magnetic field. This perturbation can be picked up by the magnetic field sensor to indicate the existence of defects.

As an alternative to the conventional eddy current testing method, velocity induced eddy currents can also be used for inspection of conductive materials [7,8,9]. In the field of nondestructive testing (NDT), velocity induced eddy current was first studied in high-speed magnetic flux leakage (MFL) testing [10,11]. A magnetizer is required to move relative to steel specimens to fulfill full scanning of the specimen surface in MFL testing [12]. At high speed, the relative movement between the magnetizer and conductors will generate considerable eddy currents to influence testing signals [12,13,14]. Afterwards, velocity induced eddy currents are used as a source to detect defects. To generate velocity induced eddy current, the excitation can be either permanent magnets [15] or a coil driven by direct current [8]. In both cases, a static magnetic field that is not varying with time is produced. To induce eddy current in the conductive material to be inspected, relative motion between the magnetic field source and the conductor is necessary. The relative motion will cause the magnetic flux of the conductor to change, and further induce eddy currents in the specimen. In order to pick up the perturbations of eddy currents due to defects, magnetic sensors are used to pick up the perturbation field [7,15,16,17,18]. Alternatively, force sensors can also be used to measure the Lorentz forces that are associated with eddy currents [18,19].

A velocity induced eddy current probe, which consists of a magnet array and a magnetic sensor, has been used to detect cracks in aluminum plates in previous studies [11]. Because the eddy current density increases with the inspection speed, the amplitudes of inspection signals for cracks also increase with inspection speed. This brings up the biggest advantage of the velocity induced eddy current testing method; that is, inspecting conductive materials in systems where there is relative motion between the probe and the material to be inspected. In addition, inspection at high speeds can be easily accomplished. Therefore, it could have potential application to the high-speed on-line inspection of aluminum and copper stripes during the manufacturing process, where a cold rolling technique is used for manufacturing. Another industry where motion is involved is the railroad industry. One of the main causes of train derailment is Rolling Contact Fatigue (RCF) cracks occurring on the surface of the rail. The proposed velocity induced eddy current probe could be attached to a train to implement the scanning of rails.

In most previous studies, the velocity induced eddy current method has been applied to aluminum. However, the field that is most in need of high-speed testing is the railroad industry. The application of the velocity induced eddy current method to the inspection of ferromagnetic materials still lacks study. In this paper, the velocity induced eddy current probe is used to detect cracks in both non-ferromagnetic and ferromagnetic metals. A brief introduction of the testing principle is given in Section 2. Then, experimental testing results for non-ferromagnetic and ferromagnetic metals are shown in Section 3. Simulations are done in Section 4 to analyze the causations and characteristics of the signals. Finally, conclusions are drawn in Section 5.

## 2. Velocity Induced Eddy Current Method

According to Faraday’s law of induction, whenever the magnetic flux through a loop changes, an electromotive force (EMF)

(1) $$\varepsilon =-\frac{d\phi }{dt}\;$$

will appear in the loop. Inside a conductor, EMF will drive the electrons to move, and accordingly eddy currents are formed. In conventional eddy current testing, an excitation coil with alternating current is placed above the conductive material to be tested. The time-varying current produces a time-varying magnetic field, hence inducing eddy current in the specimen. In the velocity induced eddy current testing method, another way of generating eddy currents is employed. The source can be either a permanent magnet or a coil excited with direct current. Although the generated magnetic field is a static field, it still generates eddy currents inside the specimen, because as the source moves, the magnetic flux in a particular region of the specimen changes, and eddy currents are induced.

The velocity induced eddy current probe discussed in this paper consists of an array of permanent magnets to produce a magnetic field, and a Hall sensor to measure it. The probe moves with a constant velocity ***v*** above the specimen surface. This problem can be considered from another perspective due to the relativity of motion. It can be viewed (in a coordinate system that is fixed with respect to the magnet) as a specimen that moves with velocity −***v*** with the magnet at rest. Then, the eddy current generated by the magnet array can be expressed as:

(2) J=−σv×B 

where ***J*** is the induced eddy current density, *σ* is the electrical conductivity of the specimen, and ***B*** is the magnetic flux density. At low speed, when the secondary field can be ignored, ***B*** represents the magnetic field produced by the magnets ***B****_M_*. The minus sign is due to the definition of the direction of velocity.

The magnetic field picked up by the Hall sensor is the vector sum of the magnetic field produced by the magnets ***B****_M_* and the magnetic field produced by the eddy currents created in the specimen ***B****_EC_*. Eddy currents have a stable path when there is no defect, and the Hall sensor picks up a constant magnetic field. The presence of a defect perturbs the eddy currents’ path, and further changes the magnetic field picked up by the Hall sensor.

At first sight, looking at the principle of velocity induced eddy current testing, it would be expected that this method could be applied to both non-ferromagnetic and ferromagnetic metals, because it seems that the only requirement is that the specimen is a conductor so that eddy currents can be generated. However, the following sections will show that the underlying principles for inspecting non-ferromagnetic and ferromagnetic metals are actually quite different.

## 3. Experimental Tests

### 3.1. Experimental Setup

**Figure 1:**
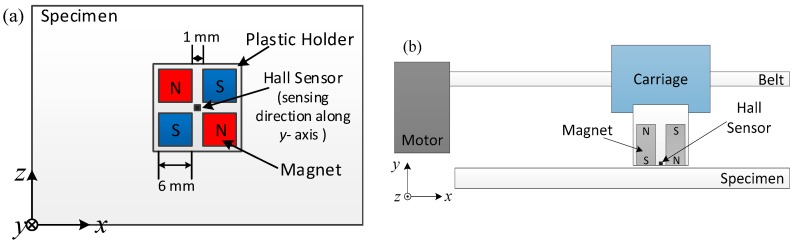
Schematic diagram of experimental setup: (**a**) Top view of probe structure; (**b**) Side view of inspection system.

Similar to the conventional eddy current testing method, the sensitivity of the velocity induced eddy current inspection system depends on the intensity of eddy currents that can be generated in specimens. With stronger eddy currents, smaller cracks can be detected. According to Equation (2), the current density is proportional to both probe velocity and the magnetic field of the magnets. In the experimental setup, an array of 4 magnets was used to create the magnetic field. The top view of the probe structure is shown in Figure 1a. The magnetization of each magnet is perpendicular to the specimen (along the *y*-axis), with neighboring magnets possessing magnetization in opposite directions. When magnets are placed close to each other, strong magnetic force will make the magnets stick together. Therefore, a plastic holder was made using a 3-D printer to fix the position of the magnets. A Hall sensor was placed in the middle of the four magnets to pick up magnetic flux density along the *y*-axis. Another important factor to generate velocity induced eddy currents is that the moving velocity of the probe should be stable and as high as possible. The inspection system is schematically shown in Figure 1b. The probe was carried by a carriage which is fixed to a belt. The belt was driven by a brushless motor, which enabled the probe to have an acceleration of 20 m/s^2^, and a maximum speed of 9 m/s.

### 3.2. Inspection of Aluminum Plates

**Figure 2:**
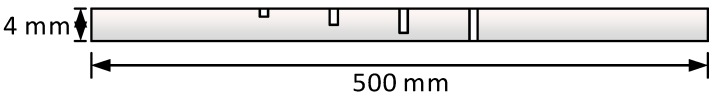
Aluminum plate used for inspection.

The aluminum plate tested is shown in Figure 2. It had four cracks with depths of 1 mm, 2 mm, 3 mm, and 4 mm. All the cracks had the same width of 0.5 mm and length of 50 mm. Four tests were conducted with different scanning speeds.

**Figure 3:**
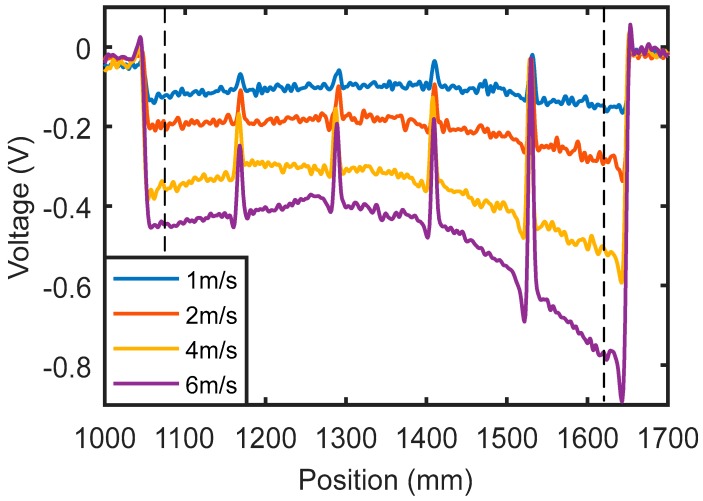
Velocity induced eddy current testing signals for cracks in an aluminum plate.

The output voltages of the Hall sensor are shown in Figure 3. The region between the two dashed lines shows the testing signals for the four cracks. The two large signals outside the two dashed lines are caused by the plate edges. From the testing results in Figure 3, all four cracks can be efficiently detected, and the signal amplitude is larger for cracks with larger depth. It can also be noticed that the signal amplitude increases with the increase in probe speed. This is a very reasonable consequence of the fact that velocity induced eddy current is proportional to the probe speed.

### 3.3. Inspection of Steel Plates

**Figure 4:**
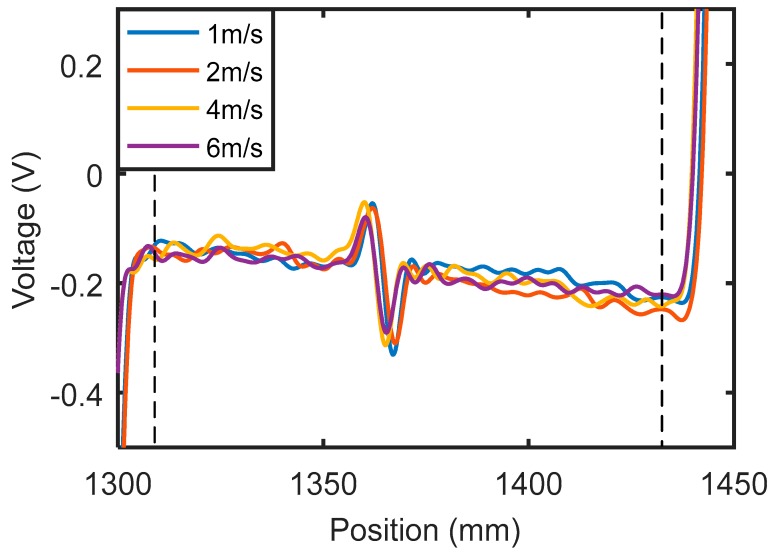
Velocity induced eddy current testing signals for cracks in a steel plate.

The same inspection system used for the aluminum plate was applied to the inspection of a carbon steel plate with thickness of 2 mm. The plate only contained a crack with depth of 1 mm. The testing results with different probe speeds are shown in Figure 4. Again, the signal between the two dashed lines is the signal for the crack, and the signals outside are caused by the plate edges. For better comparison of the signal amplitudes, a band-pass filter (with lower and higher cutoff frequencies being 20 Hz and 5 kHz) was used to eliminate high frequency noise and low frequency baseline shifts.

Unlike the situation of the aluminum plate, the signal amplitude for the crack in the steel plate did not increase with the probe speed. This was an unexpected result. According to Equation (2), the induced eddy current density is proportional to the velocity and thus the signal amplitude should increase approximately linearly with probe speed. However, the signal amplitude seems independent of probe speed from Figure 4. Therefore, it can be inferred that the signals obtained in Figure 4 are not due to velocity induced eddy current. The major difference between aluminum and steel is that aluminum is non-ferromagnetic while steel is ferromagnetic. So, in the case of the steel plate, both direct magnetic field interaction and eddy current effects should be considered. The obtained signal is the combined outcome of both effects. Besides, steel materials usually have lower conductivity than aluminum materials. At the same testing speed, the velocity induced eddy currents in a steel plate are weaker than that in an aluminum plate. So the influence of eddy current on testing signals of steel plates is less than that of aluminum plates.

## 4. Finite Element Simulations

### 4.1. Finite Element Model

In this section, finite element simulation was carried out to analyze the testing signals. The advantage of finite element simulation is that the electromagnetic properties, such as conductivity and permeability of the material, can be easily modified. Thus, the magnetic interaction and eddy current effect can be studied separately. Furthermore, the distribution of eddy currents and the magnetic field can be observed in a straightforward way.

**Figure 5:**
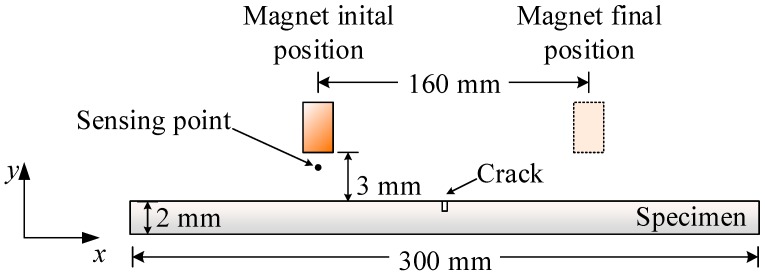
Schematics of simulation model.

The 2-D model, as shown in Figure 5, included a magnet and a plate. The magnet had width of 6 mm and height of 10 mm. Its coercivity was set to 1 MA/m, which is a typical value for neodymium (NdFeB) magnets. The thickness and the length of the plate were 2 mm and 300 mm, respectively. The magnet moved along the positive direction of the *x*-axis for a total distance of 160 mm, with fixed lift-off of 3 mm. At each time step the magnet moved 1 mm, and calculations of electromagnetic field were conducted. The magnetic flux density at the sensing point, which was 1 mm below the magnet, was extracted as a testing signal.

### 4.2. Aluminum Plates

**Figure 6:**
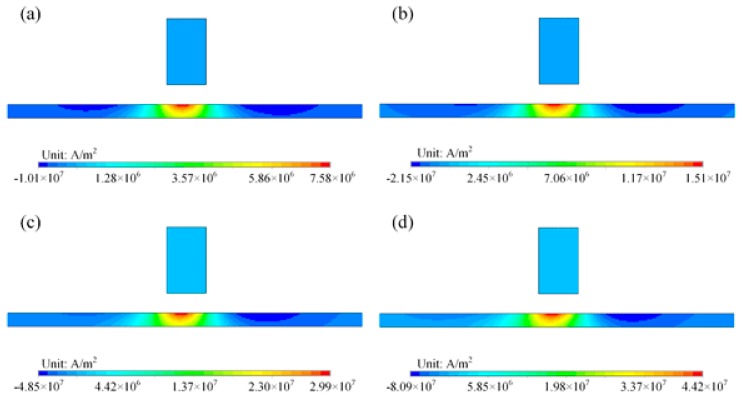
Distribution of velocity induced eddy currents in an aluminum plate for different probe speeds: (**a**) 1 m/s; (**b**) 2 m/s; (**c**) 4 m/s; (**d**) 6 m/s.

**Figure 7:**
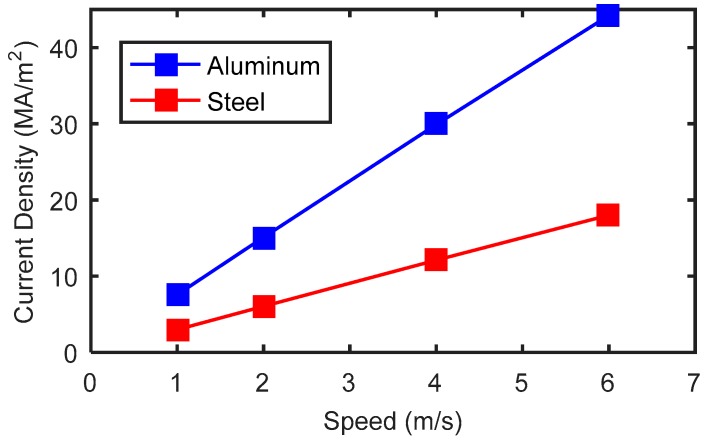
Change of maximum eddy current density against probe speed.

First, simulations were conducted for an aluminum plate without a crack. The conductivity and the relative permeability were set to 35.3 MS/m and 1, respectively. For different probe speeds, the distributions of eddy currents obtained are shown in Figure 6. The velocity induced eddy currents have a very similar pattern. They are strongest in the region beneath the magnet because this region has the largest magnetic field (*y*-component). Although the eddy currents show a similar distribution pattern, the intensities are different, as indicated in the contour legends. As shown in Figure 7, the maximum current density increases approximately linearly with magnet speed. In aluminum plates, the inspection signal is totally due to the perturbation of eddy currents. With larger eddy current density, the perturbation of magnetic field will also be larger. Therefore, the signal amplitudes increase with inspection speed, as seen in Figure 3.

### 4.3. Steel Plates

**Figure 8:**
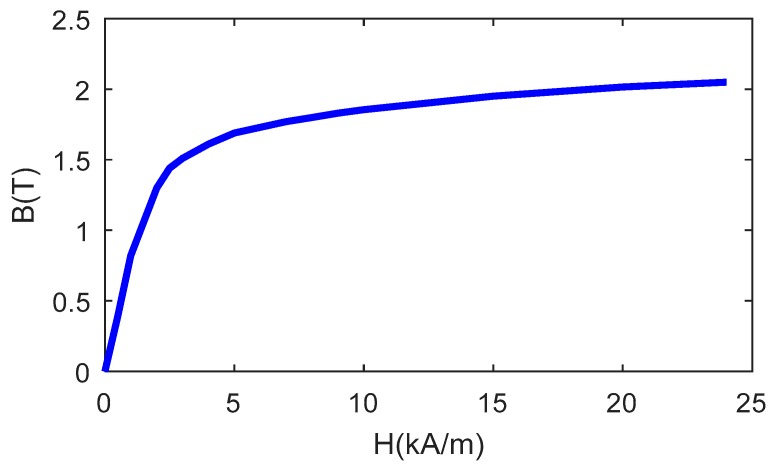
B-H curve assigned to the steel plate in simulation.

For aluminum plates, only the perturbation of eddy currents contributes to the testing signal. However, for ferromagnetic materials such as steel, the existence of defects will change the original magnetic field distribution. Therefore, two effects need to be considered: (1) Velocity induced eddy current, and (2) direct magnetic interaction between the magnet and the steel plate. The direct magnetic interaction was studied in a newly proposed NDT method called permanent magnetic field perturbation [20,21]. It was shown that when a permanent magnet moves above a steel plate, the perturbations of magnetic field caused by defects can be measured by magnetic sensors as testing signals. To consider both effects in the simulation of steel plates, a B-H curve, which is shown in Figure 8, was assigned to the steel plate, and its conductivity was set to 7.14 MS/m.

**Figure 9:**
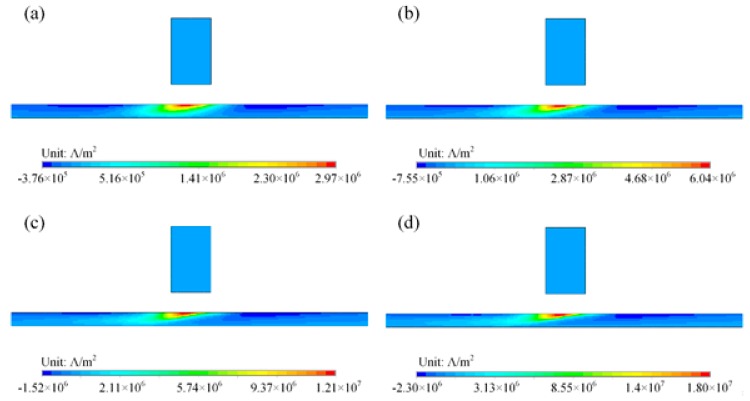
Distribution of velocity induced eddy currents in a steel plate for different probe speeds: (**a**) 1 m/s; (**b**) 2 m/s; (**c**) 4 m/s; (**d**) 6 m/s.

In a steel plate without defects, distributions of eddy currents were calculated for different probe speeds, and are shown in Figure 9. The eddy currents in a steel plate are tilted and distribute asymmetrically about the magnet. This is due to the diffusion of electromagnetic field inside the material. From Maxwell’s equations for conductors, the governing equation of an electric field can be derived as:

(3) $$\nabla^{2}E=\mu \varepsilon \frac{\partial E}{\partial t^{2}}+\mu \sigma \frac{\partial E}{\partial t}\;$$

in which, the diffusion term is:

(4) $$\nabla^{2}E=\mu \sigma \frac{\partial E}{\partial t}\;$$

Combining Equation (4) with Ohm’s law ***J*** = *σ**E***, the diffusion equation for current density can be expressed as:

(5) $$\frac{\partial J}{\partial t}=\frac{1}{\mu \sigma }\nabla^{2}J\;$$

The diffusion coefficient 1/*µσ* in Equation (5) determines how fast the diffusion process is. With a smaller diffusion coefficient, it takes longer to reach equilibrium. For the materials used in the simulation, although steel has lower conductivity *σ*, its permeability *µ* is much larger than that of aluminum. As a total effect, steel has a lower diffusion coefficient. For steel plates, when the magnet moves to the next position, the diffusion process is still going. As a result, the diffusion effect is more obvious (and causes the asymmetrical distribution of eddy currents) in steel plates, as shown in Figure 9.

The maximum values of induced eddy current density in steel plates for different speeds, with eddy currents in aluminum plates for comparison, are shown in Figure 7. The eddy current density in a steel plate also increases with probe speed. However, the eddy currents in a steel plate are weaker than that in an aluminum plate due to the low conductivity of steel.

**Figure 10:**
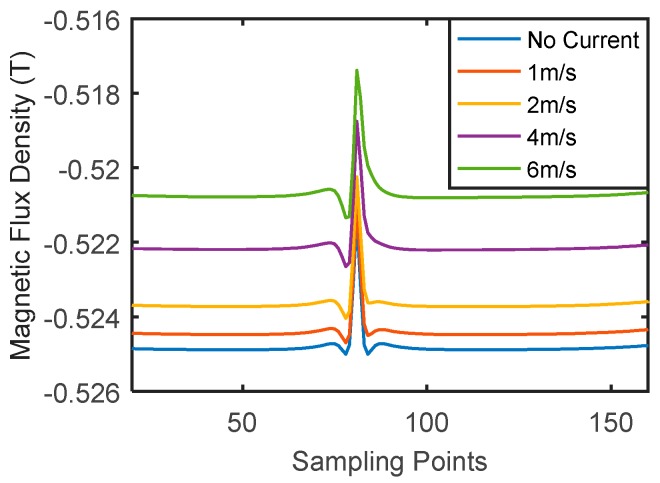
Signals obtained in a steel plate with and without eddy current effects.

Further, simulations were performed in the presence of a surface crack with depth of 1 mm and width of 0.8 mm. The *y*-component of the magnetic flux density at the sensing point was extracted as the inspection signal. The testing signals with different scanning speeds are shown in Figure 10, and the peak-to-peak amplitudes are listed in Table 1. For signals with different probe speeds, baseline shifts can be clearly seen. However, the peak-to-peak amplitudes only change by 8.1% from 1 m/s to 6 m/s. Therefore, one may conclude that testing signals are mainly due to magnetic field perturbation instead of eddy current perturbation. To further prove this statement, a simulation was performed without assigning the electrical conductivity to the steel plate. In this case, eddy currents will not be generated, and the testing signal is only due to the permanent magnetic field perturbation. The signal without eddy current is also shown in Figure 10, and its amplitude is listed in Table 1.

In conclusion, when the velocity induced eddy current probe is applied to a steel plate, the inspection signal is due to direct magnetic field perturbation rather than eddy current effects. This explains why the signal amplitudes in Figure 4 are almost independent of probe speed. Because of the band-pass filter used in experiments, baselines shifts cannot be clearly seen in Figure 4.

## 5. Conclusions

The velocity induced eddy current probe has been applied to detecting cracks in both aluminum and steel plates. The experimental results show that this probe can successfully detect cracks in both cases. With further analysis using finite element simulation, the following conclusions can be drawn:

- The velocity induced eddy current probe can detect cracks in aluminum plates. The velocity induced eddy current inside the aluminum plate is approximately proportional to the speed of the moving magnet. The inspection signal amplitude increases with crack depth and probe speed.
- The velocity induced eddy current probe can detect cracks in steel plates. The experimental results show that the signal amplitude does not increase linearly with probe speed, which means that the signal is not caused by eddy current. Two effects, namely the eddy current effect and direct magnetic field perturbation, exist when inspecting steel plates. The simulation results show that the signals obtained with and without eddy currents have almost the same amplitude, which means that the direct magnetic field perturbation is responsible for the crack detection in steel plates.

## Figures and Tables

**Table 1:** Peak-to-peak amplitudes of steel plate signals at different speeds.

Speed	Amplitude
1 m/s	3.7 mT
2 m/s	3.8 mT
4 m/s	3.9 mT
6 m/s	4.0 mT
Without eddy current	3.6 mT
